# On-Chip Circularly Polarized Circular Loop Antennas Utilizing 4H-SiC and GaAs Substrates in the Q/V Band

**DOI:** 10.3390/s24020321

**Published:** 2024-01-05

**Authors:** Rawad Asfour, Salam K. Khamas, Edward A. Ball, Jo Shien Ng, Guanwei Huang, Rozenn Allanic, Denis Le Berre, Cédric Quendo, Aude Leuliet, Thomas Merlet

**Affiliations:** 1 Department of Electronic & Electrical Engineering, University of Sheffield, Sheffield S1 3JD, UK; s.khamas@sheffield.ac.uk (S.K.K.); e.a.ball@sheffield.ac.uk (E.A.B.); ghuang4@sheffield.ac.uk (G.H.); 2 Department of Electrical Engineering, Laboratoire des Sciences et Techniques de l’Information de la Communication et de la Connaissance (Lab-STICC), University of Brest, 29238 Brest, France; rozenn.allanic@univ-brest.fr (R.A.); denis.le-berre@univ-brest.fr (D.L.B.); quendo@univ-brest.fr (C.Q.); 3 Thales LAS OME, 78990 Elancourt, France; aude.leuliet@thalesgroup.com (A.L.); thomas.merlet@thalesgroup.com (T.M.)

**Keywords:** array antenna, circular polarization, GaAs, loop antennas, millimeter wave, on-chip antennas, 4H-SiC

## Abstract

This paper presents a comprehensive assessment of the performance of on-chip circularly polarized (CP) circular loop antennas that have been designed and fabricated to operate in the Q/V frequency band. The proposed antenna design incorporates two concentric loops, with the outer loop as the active element and the inner loop enhancing the CP bandwidth. The study utilizes gallium arsenide (GaAs) and silicon carbide (4H-SiC) semiconductor wafer substrates. The measured results highlight the successful achievement of impedance matching at 40 GHz and 44 GHz for the 4H-SiC and GaAs substrates, respectively. Furthermore, both cases yield an axial ratio (AR) of less than 3 dB, with variations in bandwidths and frequency bands contingent upon the dielectric constant of the respective substrate material. Moreover, the outcomes confirm that utilizing 4H-SiC substrates results in a significantly higher radiation efficiency of 95%, owing to lower substrate losses. In pursuit of these findings, a 4-element circularly polarized loop array antenna has been fabricated for operation at 40 GHz, employing a 4H-SiC wafer as a low-loss substrate. The results underscore the antenna’s remarkable performance, exemplified by a broadside gain of approximately 9.7 dBic and a total efficiency of circa 92%. A close agreement has been achieved between simulated and measured results.

## 1. Introduction

Global mobile data traffic has increased significantly in recent years. It is expected that data traffic will continue to strain the capacity of communication networks in the future [1]. An analysis of current statistics from the International Telecommunication Union (ITU) shows that global mobile data traffic will increase to 607 exabytes (EB) per month by 2025 [2]. The exponential increase in the 5G throughput requirement drives the spectrum used for the front haul from the conventional microwave band to the millimeter wave (mmWave) spectrum. It is the consensus that the Q/V band offers numerous advantages compared to lower frequency bands. The increasing development of applications such as high-definition video streaming, mobile distributed computing, and high-speed Internet drives this preference. The unique characteristics of the Q/V band make it particularly well suited for advancing technologies and applications in these domains [3]. The Q/V band holds considerable potential that is yet to be harnessed for commercial systems. This frequency range encompasses a significant portion of the spectrum designated for satellite services, presenting promising prospects for future applications [4,5]. The main obstacle in utilizing this band is the increased propagation loss in terms of the attenuation caused by rain and the path loss [6].

Therefore, to overcome the limitations of mmWave signal propagation, the commonly used linearly polarized elements are replaced by circularly polarized counterparts [7,8]. In a parallel development, there has been considerable interest in the field of mmWave communication systems regarding circularly polarized antennas. This interest is grounded in established benefits, notably their resilience to multipath interference and their ability to tolerate misalignment between the transmitting and receiving antennas. Consequently, integrating circularly polarized antennas into mmWave designs is a strategic decision to augment the robustness and reliability of communication systems operating within these high-frequency bands. Circular polarization radiation is used in global positioning systems (GPS), satellite communications, RFID systems, ground radar, and wireless local-area networks [9]. To meet the requirements of specific applications, CP antennas can be realized by generating orthogonal modes using perturbation techniques [9]. For example, a semi-elliptical-shaped impedance transformer has been introduced into the feeding line to fine-tune the effects of electromagnetic coupling between the loop and the monopole feed [10]. On the other hand, the loop antenna offers several advantages, such as simple design and fabrication, low cost, and ease of implementation. As a result, it has been employed in many wireless applications [11].

The employment of on-chip antennas offers significant advantages in developing highly integrated transceiver systems by utilizing complementary metal–oxide–semiconductor (CMOS) technology. For example, an antenna having an artificial magnetic conductor (AMC)-based wide-slot squared design has been seamlessly integrated at 60 GHz [12]. In simulations, this integrated antenna exhibited a gain of approximately −2.1 dBi. Consequently, antenna-on-chip is promising as a viable alternative to conventional metal interconnects for connecting chips on a printed circuit board (PCB), especially when dealing with extremely wide bandwidths and sub-terahertz carrier frequencies, where traditional metal leads become impractical.

In another study, a vertical-type loop design using two wire bonds of varying sizes was introduced for 50 GHz operation using a silicon (Si) substrate [13]. These wire bonds originate from the ends of a differential line and culminate in a shorted-metal pattern. The antenna is linearly polarized, resulting in a circa 8% bandwidth return loss, along with a total efficiency of 24% and a gain of −2 dBi. In addition, the utilization of two wire bonds of differing sizes and complex feeding structures can result in a relatively large and intricate antenna configuration. Furthermore, the fabrication process may pose challenges, particularly when dealing with small dimensions, such as a 10 µm slot width and 100 µm metal-line width, demanding advanced manufacturing techniques to achieve precise dimensions at this scale. Furthermore, three on-chip antenna prototypes were designed and compared using a high-resistivity silicon substrate operating at the V band [14]. These antennas (loop, slot, and dipole) were found to have boresight gains of 3.7 dBi, 3.8 dBi, and 3.9 dBi, with corresponding radiation efficiencies of 79%, 83%, and 82%, respectively. The results highlight the effectiveness of high-resistivity substrates to significantly enhance the gain and efficiency of on-chip antennas. This demonstrates the potential of on-chip antennas as a promising technology for highly integrated millimeter wave applications. 

Other studies consider GaAs substrates, due to their higher resistivity and lower losses compared to Si substrates. For example, an on-chip antenna of a half-wavelength dipole and two tilted and slotted dipole elements was designed, using a GaAs substrate, to operate in the V-band frequency range, i.e., at 60 GHz, and the results demonstrated a gain of circa 3.6 dBi [15]. In addition, a multilayer patch antenna integrated with a V-band MMIC transmitter was fabricated on a 100 μm thin GaAs wafer and found to have a maximum gain of ~2 dBi at 57 GHz [16]. Furthermore, a 94 GHz log-periodic planar antenna has been reported, with a lapping process being utilized to reduce the substrate thickness to 100 μm for efficiency improvement purposes, resulting in an achieved gain of 4.8 dBi [17]. An on-chip antenna specifically designed for the planar coronavirus shape within the terahertz band has been introduced, with results that highlight an impressive efficiency of 93% [18].

Most of the reported on-chip antenna designs are based on utilizing Si or GaAs wafers as substrates. However, such materials suffer from relatively high dielectric losses, causing the antenna’s radiation efficiency to deteriorate at higher frequencies [19,20,21]. Additionally, the higher dielectric constants of 12 and 12.94 for Si and GaAs substrates, respectively, may result in a stronger surface wave excitation for a given wafer thickness. On the other hand, 4H-SiC offers lower dielectric losses compared to Si and GaAs substrates as well as having the distinctive advantage of the capability to work in harsh environments, such as high-temperature and high-power environments [22], which makes it most suitable for the design of conventional antennas and antenna sensors that operate in such conditions. Despite the considerable number of reported studies on the potential of 4H-SiC-based devices, only a few studies are available on the design of 4H-SiC-based antennas [23,24]. For example, a patch antenna has been fabricated on a 4H-SiC substrate operating at 10 GHz, resulting in narrow impedance bandwidth and gain of 2 dBi [23]. On the other hand, a simulations-based study was reported in [24] to demonstrate the potential of a mmWave dielectric resonator antenna on a 4H-SiC substrate.

This paper presents the design and comprehensive measurements of a circularly polarized on-chip loop antenna fabricated on a 4H-SiC substrate to operate in the Q/V frequency band. This design delivers commendable performance characteristics in terms of both return losses and axial-ratio bandwidths. In addition, for comparison purposes, two different substrates have been considered, namely 4H-SiC and GaAs. The performance of a single-loop antenna on each substrate has been thoroughly examined, focusing on key parameters such as return losses, circular polarization, gain, and radiation efficiency. Furthermore, a 4-element 4H-SiC-based array has been designed and found to have a high gain of 9.7 dBic. In the proposed array design, a parallel feed network has been implemented, which plays a crucial role in optimizing the antenna’s performance. Different circular polarization senses can be attained by changing the position of the gaps in the loop antenna. This capability enables the antenna to produce various types of circular polarization, enhancing its versatility and adaptability for different communication needs. The simulations were conducted using the Computer Systems Technology (CST 2020) Microwave Studio software. A close agreement was achieved between simulations and measurements. This research contributes to the development and understanding of 4H-SiC-based on-chip antennas for Q/V-band frequencies, showcasing their potential applications in various wireless communication and sensing systems, such as millimeter-wave wireless sensing networks. In addition, on-chip CP antennas and arrays fabricated on 4H-SiC substrates have not been reported previously. Additionally, a comprehensive comparison between the performances of antennas on GaAs and antennas on 4H-SiC substrates demonstrates the superior performance of the 4H-SiC-based antennas operating in the Q/V band.

The paper is organized as follows: Section 2 introduces the configuration and design principles of a circularly polarized on-chip antenna on a GaAs substrate. The measurements are presented in Section 3 with a detailed investigation of the CP radiation options. This GaAs-based prototype is used as a reference against which the performance of a 4H-SiC-based antenna is compared, in Section 4, through fabrication and measurement. Section 5 outlines the methodology for the proposed on-chip array antenna design using a 4H-SiC substrate and covers the prototype development, simulation, fabrication, and measurement of the proposed antenna array. The paper is concluded in Section 6.

## 2. Antenna Configuration

This section presents the proposed single antenna on two different substrates. For the design of a single element, three layers are used, including the actual antenna, a GaAs or 4H-SiC substrate having a thickness of 350 μm, and a copper ground plane measuring 10 mm × 10 mm.

**Figure 1:**
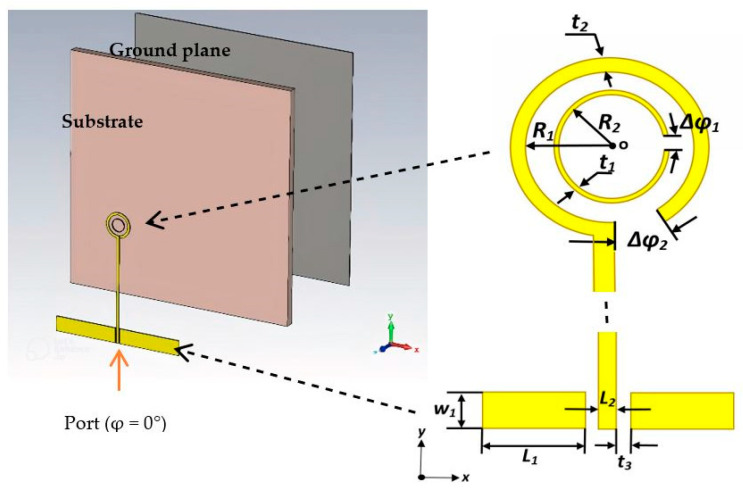
The geometry of the proposed on-chip circular loop antenna.

Figure 1 depicts the configuration of the utilized CP antenna element, and Table 1 lists the optimized geometrical parameters of the CP loop antennas. The printed circular loop and substrate are positioned above a square copper plate, which functions as a reflector. The proposed antenna comprises two concentric loops, with the outer loop as the active element. To achieve the CP radiation, the outer loop’s circumference needs to be approximately one effective wavelength and a gap needs to be introduced along the loop. With the aid of Equations (1)–(3), the circumference of the outer loop can be calculated as [25]

*ε_eff_* ≈ (*ε_r_* + 1)/2. (1)

*λ_eff_* = *λ*_0_/√(*ε_eff_*). (2)

2π*R*_1_ ≃ *λ_eff_* (3)

where *ε_eff_* is the effective relative permittivity, *λ_eff_* is the effective wavelength, and *λ*_0_ is the free-space wavelength.

**Figure 2:**
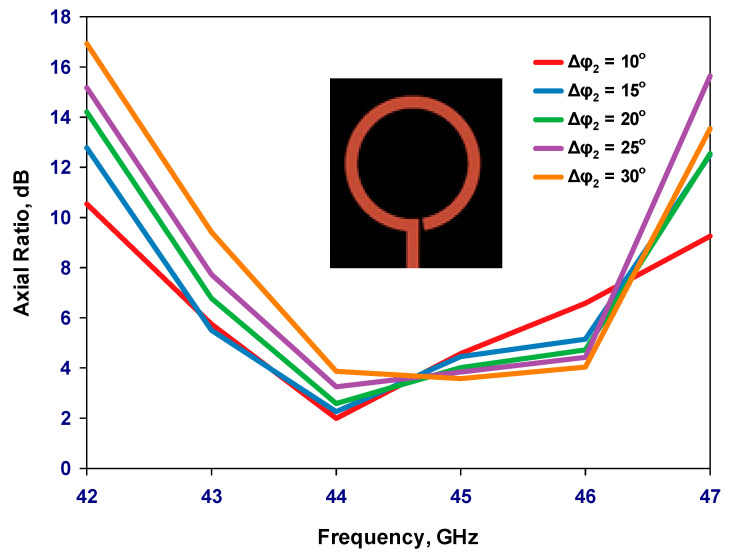
Simulated axial ratio versus frequency as a function of the outer loop’s gap.

**Figure 3:**
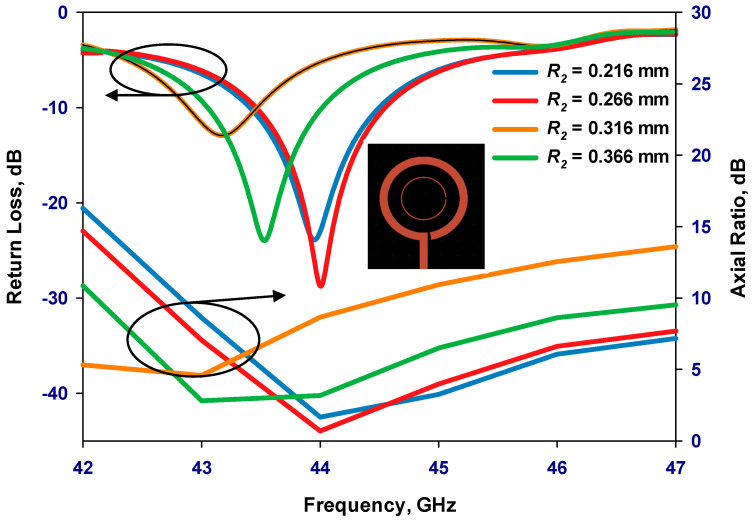
Simulated return losses and axial ratio versus frequency for different radii of the inner loop.

**Figure 4:**
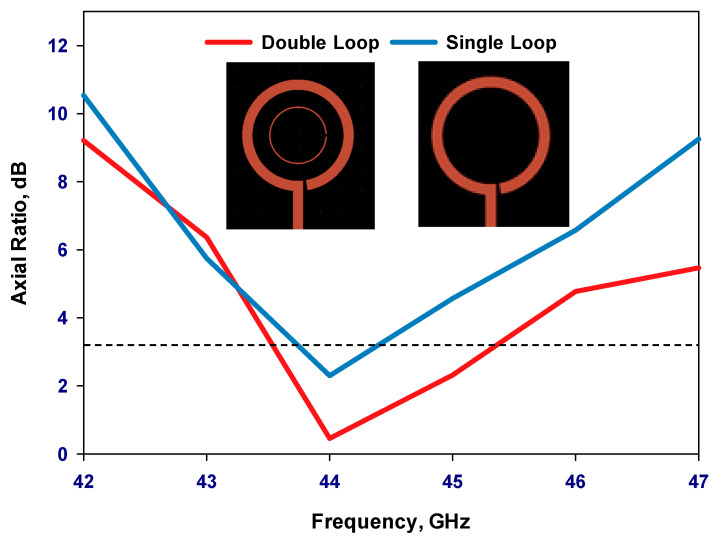
Simulated axial ratio versus frequency for the single and double loop antennas.

**Figure 5:**
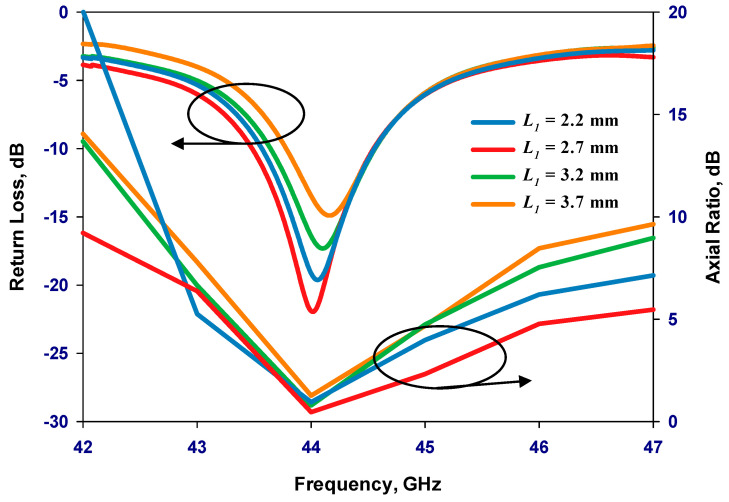
Simulated return losses and axial ratio versus frequency for different lengths of pads.

Once the outer loop’s circumference is determined, the gap has been optimized to create an optimal distribution of the traveling-wave current along the loop, which is crucial for achieving circular polarization. The antenna has been designed for operating frequencies between 40 and 44 GHz. By adjusting the size of the Δφ_2_ gap, the required axial ratio can be achieved. Additionally, the inner loop’s radius has been optimized for the widest AR bandwidth. Finally, the feeding pads have been adjusted for optimum performance. The parametric study is depicted in Figure 2, Figure 3, Figure 4 and Figure 5 for the GaAs-based antenna. Figure 2 showcases the variation in the axial ratio for various values of Δφ_2_ to identify the most favorable angular gap that yields the widest axial-ratio bandwidth in the absence of the inner loop. As a result, the optimum Δφ_2_ has been determined as 10°. In Figure 3, the inner loop’s radius undergoes variation, ranging from 0.166 mm to 0.366 mm, while keeping the outer loop radius constant at 0.441 mm. The axial ratio’s response to these changes reveals optimal performance when the inner loop’s radius is set to 0.266 mm. Figure 4 presents the simulated axial ratio, comparing two scenarios: with and without the parasitic loop. Upon integrating the inner loop, the AR ≤ 3 dB bandwidth experiences a notable increase from 1.55% to 4.75%, which is in line with CP bandwidths achieved at lower frequencies [10]. As anticipated, the parasitic loop significantly enhances the AR bandwidth, given that each loop generates an AR minimum. The merging of these two minimum AR points results in the observed bandwidth improvement. Figure 5 illustrates a parametric analysis of the pad size, offering a detailed comparison of the proposed antenna’s return losses and axial ratio performance when paired with different pad lengths. Consequently, a pad length of approximately 2.7 mm has been chosen.

**Figure 6:**
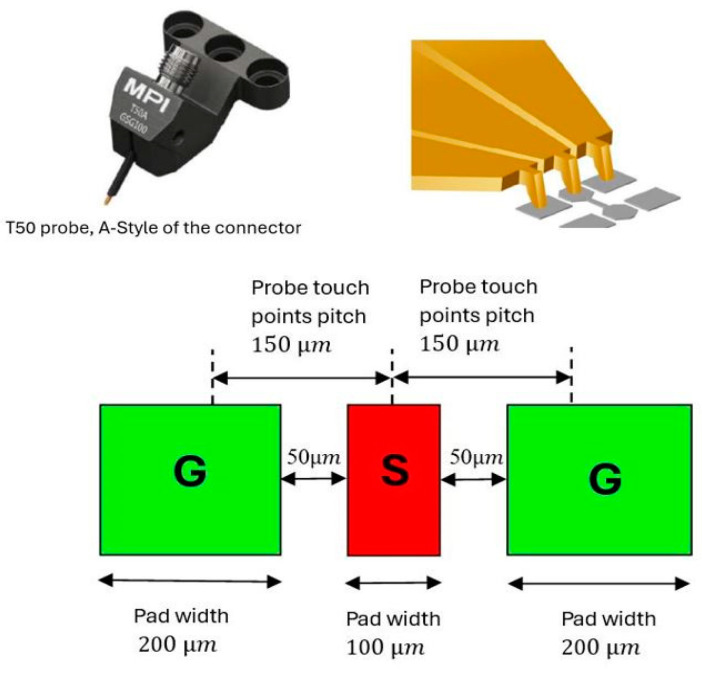
RF probes and landing-pad dimensions.

Furthermore, due to the physically small antenna size at high frequencies, a coplanar waveguide feed using ground–signal–ground (GSG) contact geometry has been utilized for measurements, as illustrated in Figure 6. The center-to-center spacing between the probe tips is 150 μm. The width of the feed line, *l*_2_, has been set at 100 μm. The distance between the feed line and the grounded rectangular pads, *l*_3_, has been set at 50 μm for optimum 50 Ω matching.

## 3. GaAs-Based Antennas with Different Polarization Senses

**Figure 7:**
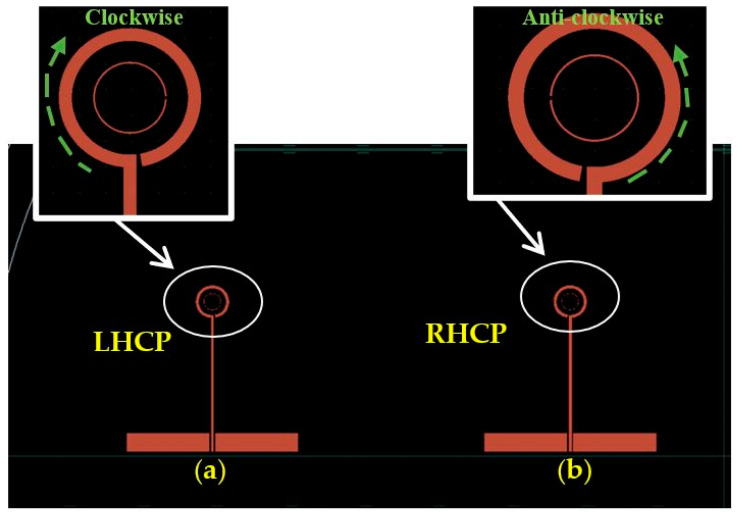
The configuration of various polarization modes for a GaAs antenna operating at 44 GHz with (**a**) LHCP and (**b**) RHCP.

**Figure 8:**
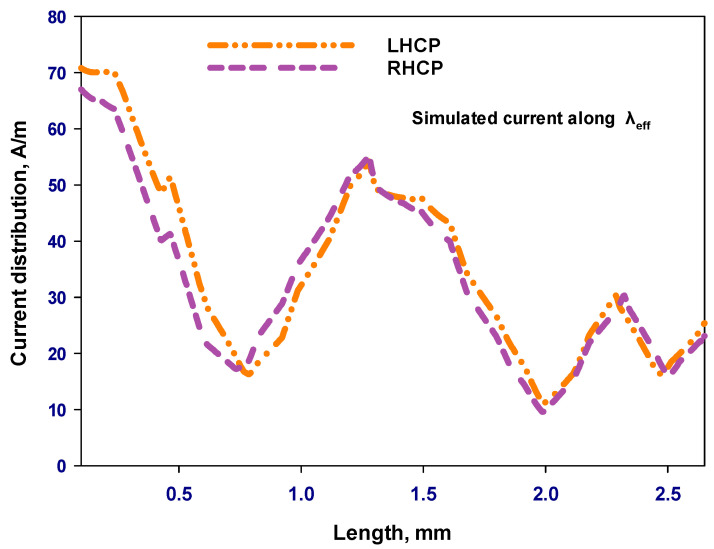
Simulated current distribution along a loop on a GaAs antenna at 44 GHz, for LHCP and RHCP.

**Figure 9:**
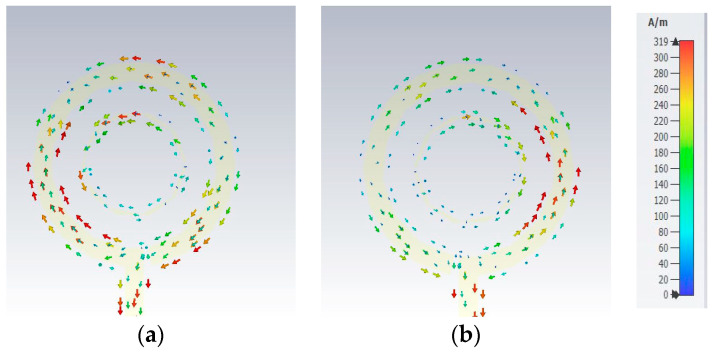
Simulated current distributions at 44 GHz for (**a**) LHCP and (**b**) RHCP.

Figure 7 presents two on-chip GaAs-based antennas that are designed to provide CP radiation at 44 GHz. These antennas exhibit two different types of polarization, a phenomenon that is accomplished through the strategic placement of the gaps. It is worth noting that a loop antenna having one effective wavelength circumference inherently radiates a linearly polarized wave. However, the introduction of a gap within a circular loop antenna induces a traveling-wave current, leading to the generation of circularly polarized radiation. When the gaps Δφ_1_ and Δφ_2_, for the outer and inner loops, are positioned on the right-hand side of the antenna structure, as illustrated in Figure 7a, they foster the formation of a traveling-wave current that is circulating in a clockwise direction, resulting in a LHCP wave. Conversely, in the antenna configuration portrayed in Figure 7b, i.e., when the gaps of Δφ_1_ and Δφ_2_ are situated on the left-hand side, the generation of an RHCP wave is facilitated. Figure 8 presents the simulated current distribution along a loop having a circa one-wavelength circumference at 44 GHz. In contrast to linear polarization, where the current maintains a standing wave distribution throughout the ring’s length, the traveling-wave current progresses along the loop, and its amplitude undergoes gradual fluctuations, diminishing close to the gap. Figure 9a,b illustrate the outcomes of CST simulations that depict how the current flows within a loop antenna when it operates in both LHCP and RHCP modes. Simulations help in understanding the antenna’s behavior and performance under different polarization requirements.

**Figure 10:**
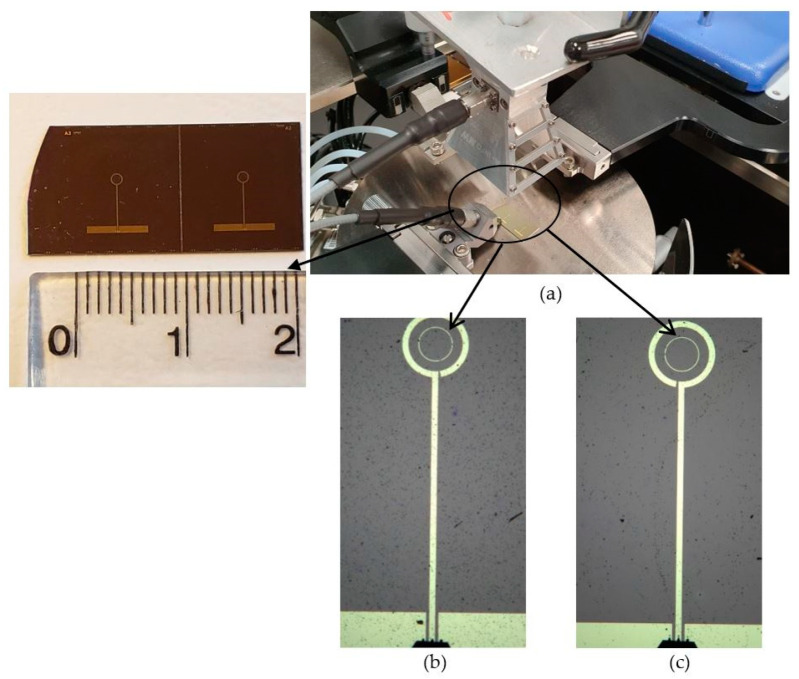
The fabricated prototype of the on-chip GaAs antenna at 44 GHz. (**a**) Antenna under test, (**b**) LHCP antenna, and (**c**) RHCP antenna.

Figure 10 presents the fabricated prototype of the on-chip GaAs antennas under test for different polarization senses, operating at 44 GHz. The antennas are based on a 350 μm thick semi-insulating GaAs substrate. A 240 nm silicon oxide was deposited on the substrate to form a dielectric layer via plasma-enhanced chemical vapor deposition (PECVD). To ensure the adhesion between the dielectric layer and the metal, a standard pressure (900 mTorr) recipe of PECVD was chosen for the SiO_2_ deposition. This was followed by a standard photolithography process, in which Ti/Au having a thickness of 40/400 nm was deposited to effect metallization of the antenna and the ground plane using the thermal evaporation technique.

**Figure 11:**
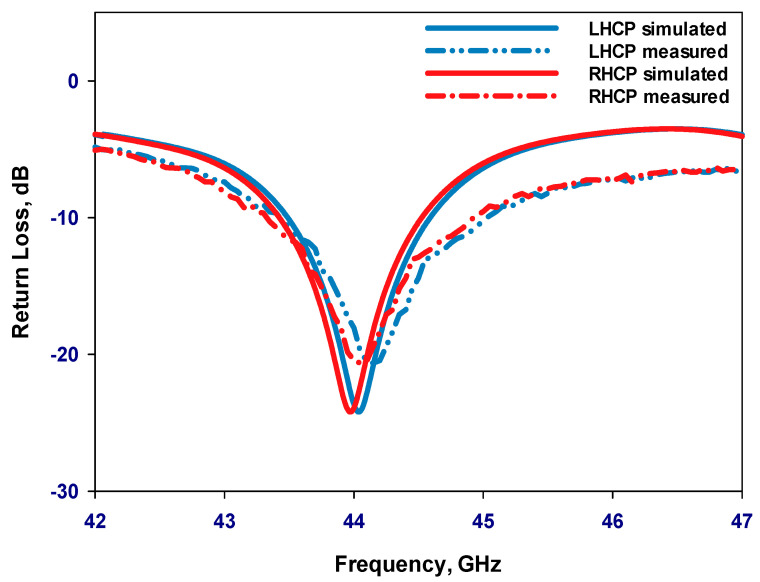
Reflection coefficients of the on-chip antenna on the GaAs substrate.

**Figure 12:**
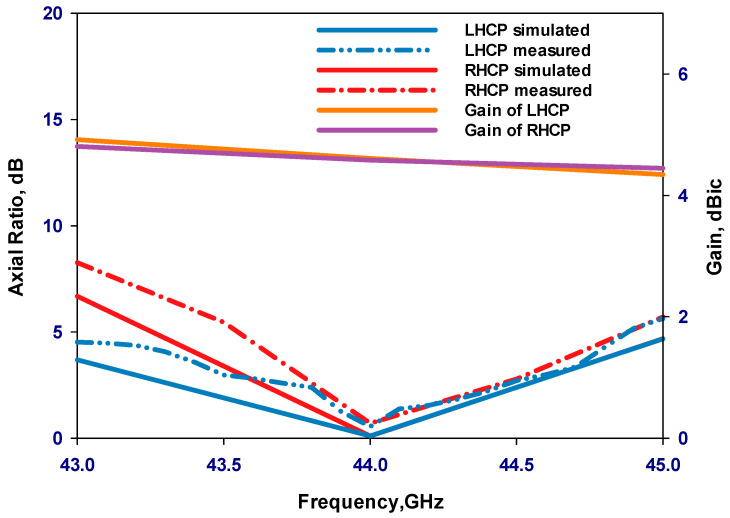
Axial ratio and gain for both circular polarization senses in the main-beam direction.

As depicted in Figure 11, there is a notable concurrence between the experimental reflection coefficients and their simulated counterparts. The measured outcomes demonstrate an impedance bandwidth of 3.68% for the LHCP state and 3.7% for the RHCP state. It is worth noting that the simulated S_11_ bandwidths for both LHCP and RHCP are approximately 3.6%, affirming the consistency between experimental and simulated results. Figure 12 presents the measured and simulated axial ratio for the LHCP and RHCP states, demonstrating an agreement between the measured and simulated AR bandwidths. In evaluating the bandwidth characteristics of CP antennas, the overlapping bandwidth between impedance and AR bandwidths is typically considered. When comparing Figure 11 and Figure 12, it becomes evident that the measured overlapped bandwidths for the RHCP and LHCP states are approximately 2.75% and 2.8%, respectively, while the simulation results indicate overlapped bandwidths of around 3.2% for the RHCP and 3.4% for the LHCP states. Figure 12 also displays the simulated realized gain of the GaAs antenna proposed for operation at 44 GHz. It is noteworthy that the simulated realized gain is consistent at circa 4.6 dBic for both polarizations. The presented investigation reveals excellent agreement between the simulation and measurement results at the operating frequency.

## 4. Performances of the 4H-SiC-Based Antenna

**Figure 13:**
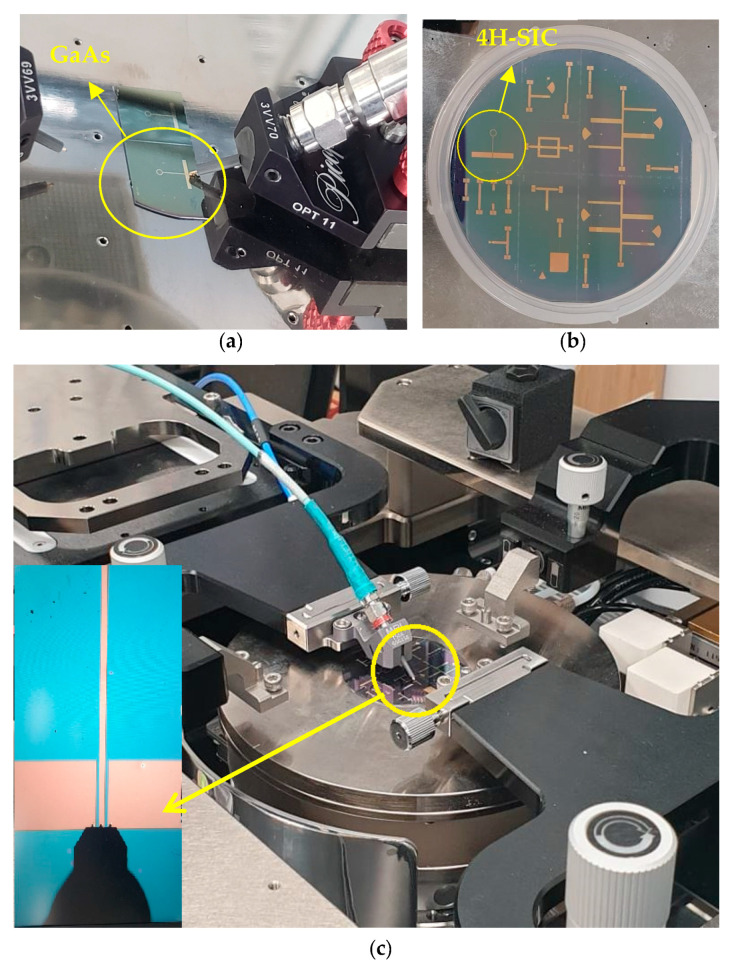
The single-loop antenna within the testing environment (**a**) on the GaAs substrate, (**b**) on the 4H-SiC substrate, and (**c**) under test using an RF wafer probe.

Images of the measurement process are depicted in Figure 13, which illustrates the single-loop antenna within the testing environment using the two substrates: GaAs and 4H-SiC. Limitations in the manufacturing process are associated with the width of the lines, and it is feasible to reduce them to 5 μm without encountering any significant issues. In addition, 4H-SiC substrates come at a higher cost than GaAs substrates. Furthermore, during the research phase, the expenses associated with device fabrication, involving ion implantation and thermal annealing, are also elevated for 4H-SiC compared to GaAs. This higher cost is attributed to the increased thermal annealing temperature necessary for 4H-SiC. In contrast, the GaAs substrate exhibits greater fragility in comparison to 4H-SiC. Consequently, the GaAs substrate could potentially have lower reliability in harsh testing environments. On the other hand, at millimeter and terahertz frequencies, the higher cost of 4H-SiC-based systems can be compensated for by lower substrate losses, which result in more efficient on-chip antennas and arrays having smaller footprints and hence drive the overall cost down. Moreover, the cost-effectiveness can be higher when the on-chip antennas are integrated with active 4H-SiC elements such as PIN diodes that offer lower insertion and switching losses compared to their GaAs counterparts [26]. Since only passive structures are considered in this study, a moderate power of circa 20–30 dBm can be easily handled. However, the utilized N5245B vector network analyzer (VNA) offers a maximum output power of 13 dBm.

**Figure 14:**
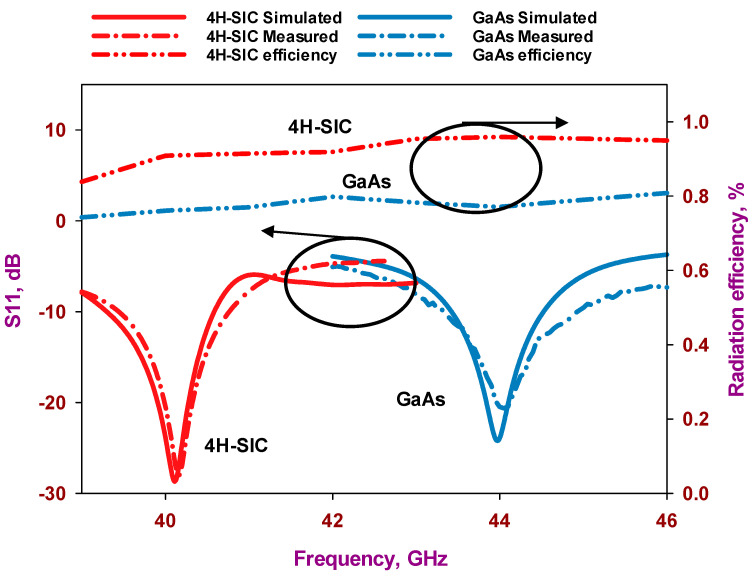
Return losses and radiation efficiencies of the two on-chip antenna configurations.

Figure 14 presents a comparison between the measured and simulated return losses for the proposed single element when fabricated on the 4H-SiC and GaAs substrates. The results show that the antennas using 4H-SiC and GaAs substrates exhibited measured S_11_ bandwidths of approximately 3.5% and 3.6%, respectively, which agrees closely with the simulations. These bandwidths extend over frequency bands of 39.4–40.8 GHz for the 4H-SiC-based antenna and 43.35–44.93 GHz for the GaAs-based counterpart, which correspond to respective bandwidths of 3.68% and 3.6%. It should be noted that different frequency bands have been achieved owing to the different electrical thicknesses of the GaAs and 4H-SiC substrates. Moreover, the physical dimensions of each loop antenna were determined using the effective wavelengths, which are based on approximations using Equation (1). It is noteworthy that Figure 14 also presents the radiation efficiencies, and it can be observed that the GaAs-based configuration offers a radiation efficiency of 78%, which is notably lower than that of ~95% for the 4H-SiC-based antenna. This clearly demonstrates the lower losses of the 4H-SiC substrate, which results in higher radiation efficiency.

**Figure 15:**
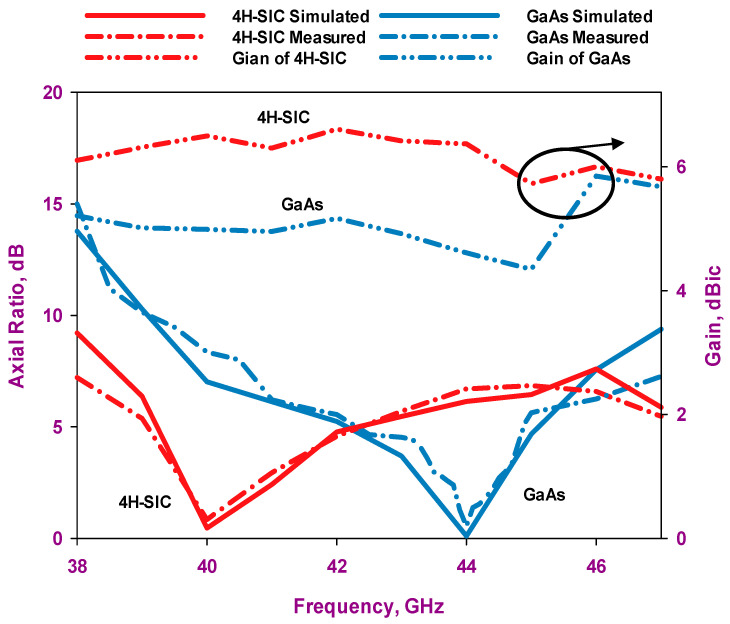
Gain and axial ratio of the two on-chip antenna configurations.

**Figure 16:**
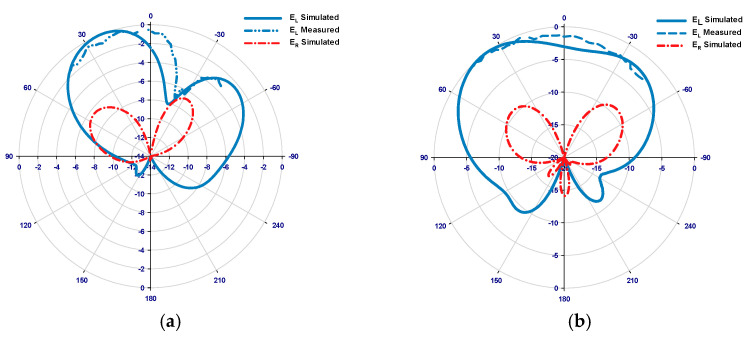
E_L_ and E_R_ radiation patterns for the on-chip loop antenna using (**a**) the 4H-SiC system and (**b**) the GaAs system.

Figure 15 illustrates the measured and simulated axial ratios as well as the simulated gains of the proposed antennas. The results reveal that the measured AR bandwidths for the 4H-SiC-based and GaAs-based configurations are 4.75% and 2.8%, respectively. In comparison, the respective simulated AR bandwidths are 4.75% and 3.4% for the 4H-SiC-based and GaAs-based antennas. Additionally, a narrower bandwidth is achieved when the GaAs substrate is utilized, which can be attributed to the higher dielectric constant of this substrate. Furthermore, the simulated single antenna’s gains for 4H-SiC and GaAs configurations are ~6.5 dBic and 4.6 dBic, respectively. Once more, the highest broadside gain has been achieved by utilizing the 4H-SiC substrate and is in line with the higher radiation efficiency, which is attributed to the lower loss tangent and weaker surface waves created by the substrate with lower permittivity. Figure 16a,b present the simulated and measured radiation patterns for the proposed antenna within the principal plane, ϕ = 0°, at 40 GHz and 44 GHz, corresponding to 4H-SiC and GaAs configurations, respectively. The patterns reveal the generation of an LHCP wave, since E_L_ is larger than E_R_, as demonstrated for both configurations. This observation also signifies that a clockwise traveling-wave current circulates along the surface of the loop antenna.

## 5. Performances of the 4H-SiC-Based Array

**Figure 17:**
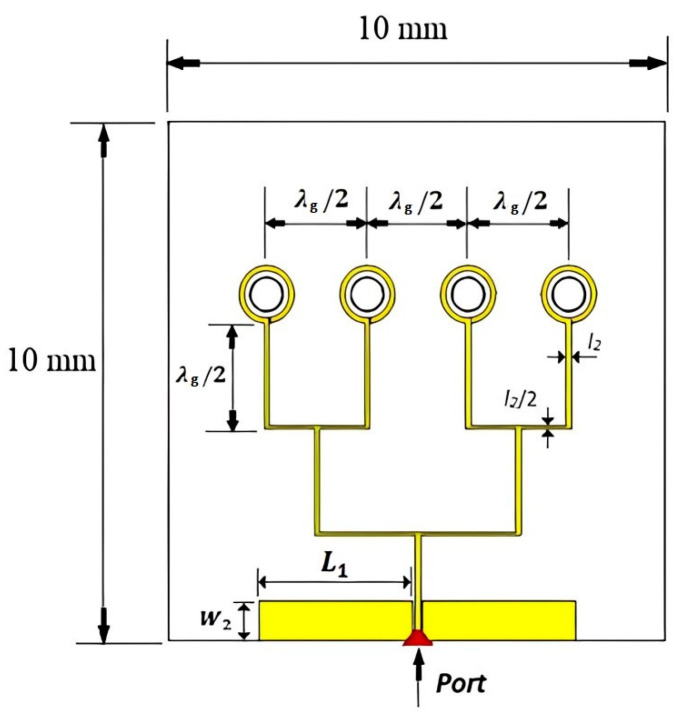
The proposed configuration of the on-chip antenna array using the 4H-SiC substrate.

Following the successful testing of a single on-chip CP antenna, the investigations were extended to design a 4-element on-chip antenna array employing a 4H-SiC substrate having dimensions of 10 mm × 10 mm × 350 μm. Building upon the performance insights gained from the individual antennas, we proceeded to design a 1 × 4 uniform linear antenna array having a precise spacing of 1.9 mm between adjacent elements, which is approximately equivalent to λ_g_/2 at 43 GHz. The antenna spacing was chosen to achieve maximum gain with minimum side lobe levels (SLLs). The antenna array is fed via uniform-length feeding lines for all elements using a 0.1 mm wide microstrip transmission line, as presented in Figure 17. This design choice ensures uniform signal amplitudes and phases across all elements, resulting in constructive interference and the formation of a robust main beam directed at θ = 0°.

**Figure 18:**
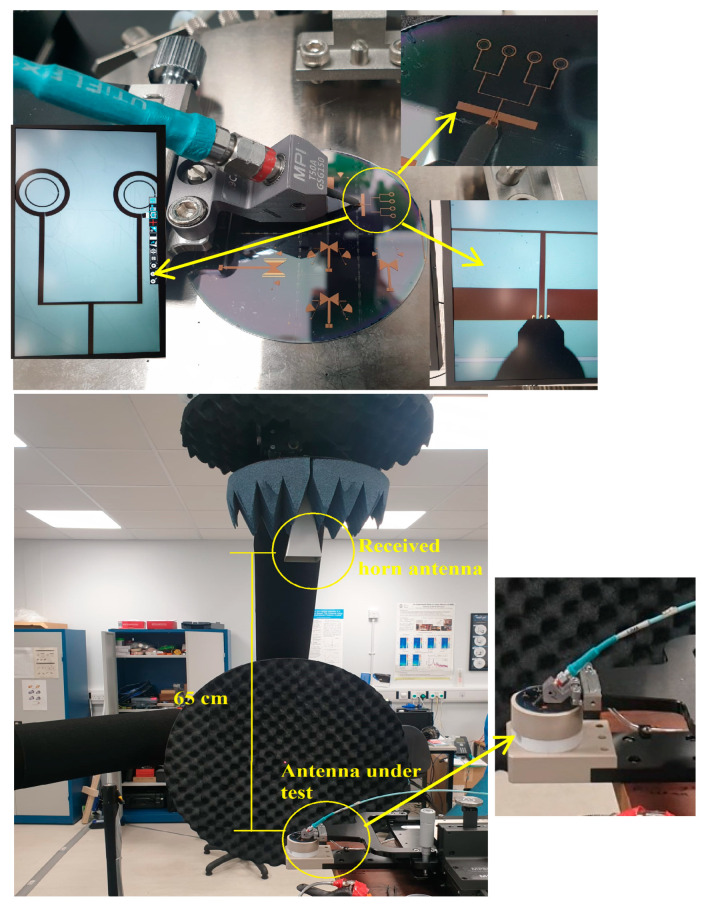
The array antenna in the mmWave measurements lab [27].

**Figure 19:**
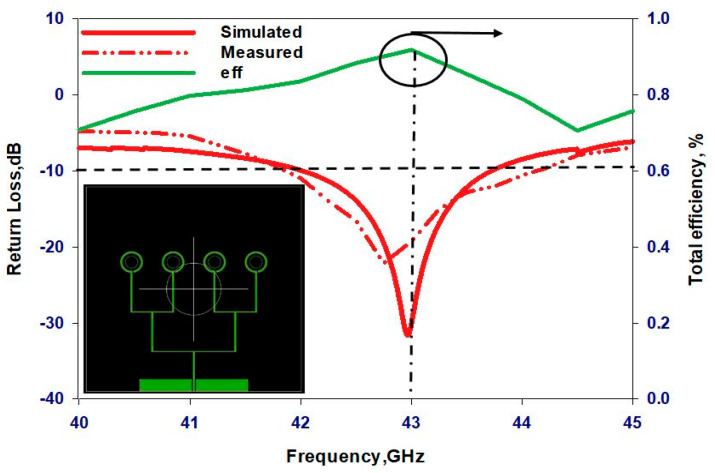
Reflection coefficients and total efficiency of the on-chip array using the 4H-SiC substrate.

**Figure 20:**
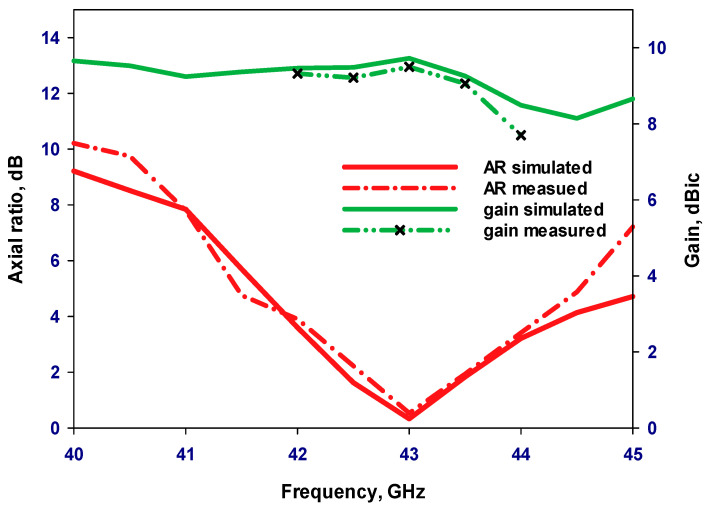
Axial ratio and gain of the on-chip array antenna using the 4H-SiC substrate.

**Figure 21:**
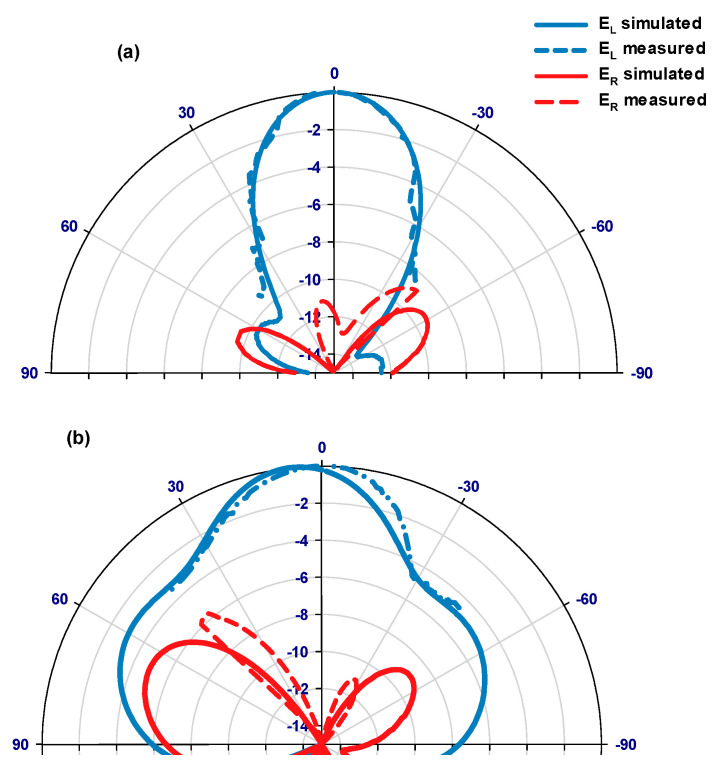
The radiation patterns of E_L_ and E_R_; at (**a**) ϕ = 0°, and (**b**) ϕ = 90° for the on-chip array on the 4H-SiC substrate.

Figure 18 presents the measurements setting of the Q/V-band circularly polarized array antenna within the testing environment. The restriction in measuring the on-chip antenna within a range of ±45° is attributed to the limitations of the measurement equipment, specifically the obstructed movements of the mmWave field measurement probe caused by the presence of the wafer prober. Beyond this range, an unobstructed view of the die becomes impractical due to interference from the metalwork of the probes. Figure 19 presents the simulated and measured return losses as well as the simulated total efficiency of the 4H-SiC-based antenna array. These results show that the antenna array boasts simulated and measured impedance bandwidths of approximately 4.4% and 4.6%, respectively. The array impedance bandwidth is slightly wider than that of a single antenna due to mutual coupling between elements. Furthermore, total efficiency has reached approximately 92%. The simulated and measured axial ratio and gain of the proposed array are presented in Figure 20. The simulated 3 dB AR bandwidth is approximately 4%, which is in close agreement with the measured counterpart of 3.9% centered at 43 GHz. Furthermore, the realized gain, as measured and simulated, is approximately 9.7 dBic and 9.6 dBic, respectively, at 43 GHz. It is worth noting that the 4H-SiC array exhibits a 3 dB gain advantage compared to the single element. It is important to emphasize that the relationship between the number of elements and the gain is not strictly linear. The addition of more antenna elements may not yield a commensurate increase in gain, primarily because the radiation pattern’s main beam may not be significantly narrower for each additional element. Furthermore, another critical consideration for achieving only a 3 dB gain enhancement in the array is the limitation imposed by the constrained size of the ground plane, which matches that of the single-element antenna. Expanding the ground plane dimensions presents itself as a viable strategy for augmenting antenna gain. Figure 21 depicts the measured and simulated E_L_ and E_R_ radiation patterns at both ϕ = 0° and ϕ = 90° at 43 GHz. Notably, there is close agreement between the measured and simulated patterns. In this case, the electric field component E_L_ surpasses E_R_ by more than 15 dB, indicating the generation of an LHCP wave.

Table 2 presents a comparative analysis between the suggested antenna and prior investigations that utilized Si and GaAs wafers as substrates. It is evident from this comparison that the 4H-SiC-based antenna exhibits superior characteristics, including increased gain and higher efficiency in combination with circular polarization. In addition, the 4H-SiC-based antenna offers a considerably higher broadside gain.

## 6. Conclusions

It is well known that the performance of on-chip loop antennas operating at high frequencies can deteriorate owing to dielectric and surface wave losses. Consequently, the selection of an appropriate substrate having the right dielectric constant and loss tangent plays a crucial role in achieving optimal antenna performance. In a comparative study, the circular loop antenna is designed and fabricated to provide CP radiation utilizing two different dielectric substrates, GaAs and 4H-SiC, operating within the Q/V frequency band. The potential for designing and fabricating an on-chip mmWave antenna on a 4H-SiC substrate has been demonstrated for the first time. The results demonstrate that using a 4H-SiC substrate, with its lower dielectric constant and loss tangent, offers the highest gain, reaching 6.5 dBic, and a high efficiency of circa 95% for the single antenna configuration. As a result, a 4-element CP array antenna employing a 4H-SiC substrate in the Q/V band was successfully fabricated and measured. The results are highly promising, with the on-chip antenna array exhibiting an impressive gain of approximately 9.7 dBic combined with a commendable total efficiency of 92%. Furthermore, the antenna array achieves left-hand circular polarization and offers an axial-ratio bandwidth of circa 4%. The achieved results demonstrate an excellent agreement between simulations and measurements. Additionally, the results indicate that a particularly good impedance matching was achieved.

In addition, the benefits of using a low-loss 4H-SiC substrate compensate for the higher cost and pave the way to design highly efficient and compact mmWave and THz antennas that meet the requirements of modern communication systems. The focus of this study was on the significance of the low losses of 4H-SiC substrates in the Q/V frequency band, and it can be extended to investigate the 4H-SiC-based antenna’s performance when operating in a harsh environment.

## Figures and Tables

**Table 1:** Parameters of the Antennas; length Dimensions are in mm.

Symbol	Quantity	GaAs	4H-SiC
ε_r_	relative permittivity	12.94	10.2
tan *δ*	loss tangent	0.006	0.00003
*R*_1_	outer loop radius	0.441	0.55
*R*_2_	parasitic loop radius	0.266	0.34
*t*_1_	outer loop width	0.1	
*t*_2_	parasitic loop width	0.014	0.02
*L*_1_	pad length	2.69	3.18
*l*_2_	transmission line width	0.1	
*l*_3_	gap between the transmission line and pads	0.05	
*w*_1_	pad width	0.574	0.85
*w*_2_	length between the pads and outer loop	3.886	
*a*_0_	antenna thickness	0.035	
*h*	substrate thickness	0.35	
*t*	reflector thickness	0.035	
Δφ_1_	parasitic loop gap	5°	
Δφ_2_	outer loop gap	10°	

**Table 2:** Performance comparison of the on-chip antenna designs using different substrates.

Ref.	Antenna Type	Number of Elements	Substrate	Frequency GHz	S_11_ BW%	Polarization	AR BW%	Gain	Efficiency %
[14]	Vertical-type wire loop	1	Silicon	50	⸙ 8	LP	**	−2 dBi	24
[15]	Slot loop	1	Silicon	60	**	LP	**	−2.1 dBi	**
[16]	Slot, dipole	1	Silicon	70	⸙ ~8.5	LP	**	3.7 dBi, 3.9 dBi	79, 82
[17]	Dipole based	1	GaAs	61	⸙ 11.6	LP	**	3.6 dBi	**
[18]	Patch	1	GaAs	57, 59	**	LP	**	−1.5 dBi, ~1 dBi	33, 21
[19]	Log periodic	1	GaAs	94	12.5	LP	**	4.8 dBi	**
[28]	Dipole	4	SiGe	250	**	CP	⸙ ~1.4	⸙ ~−9 dBi	30
[29]	Concentric ring	1	Silicon	24	35	CP	15.2	−4.5 dBi	**
[30]	Patch	1	Silicon	60	4.7	CP	1	0.3 dBi	25
This work	Open loop	1	GaAs	44	3.6%	CP	3.4	4.6 dBic	78
This work	Open loop	1	4H-SiC	40	3.5%	CP	4.75	6.5 dBic	95
This work	Open loop	1 × 4	4H-SiC	43	4.6%	CP	4	9.7 dBic	92

** The values were not supplied. ⸙ The exact values were not supplied, and the data were obtained by extrapolating the given information.

## Data Availability

Data are contained within the article.
